# Beyond the Numbers: Reframing Uric Acid Elevations in Bempedoic Acid Therapy

**DOI:** 10.1016/j.jacadv.2025.102482

**Published:** 2025-12-19

**Authors:** Federica Fogacci, Claudio Borghi, Arrigo F.G. Cicero

**Affiliations:** a Hypertension and Cardiovascular Risk Research Center, Medical and Surgical Sciences Department, Alma Mater Studiorum University of Bologna, Bologna, Italy; b Cardiovascular Medicine Unit, Heart, Chest and Vascular Department, IRCCS Azienda Ospedaliero-Universitaria di Bologna, Bologna, Italy

We read with great interest the brief report by Prof. Ray et al on the association between uric acid–lowering therapies and gout incidence among patients treated with bempedoic acid in the CLEAR (Cholesterol LowEring via bempedoic acid, an ACL-inhibiting Regimen) Outcomes trial.^1^ The authors are to be commended for addressing an often-overlooked metabolic aspect of bempedoic acid therapy and for providing valuable insights into the management of hyperuricemia in this population. We would like to offer a few considerations that may further contextualize and expand the clinical interpretation of these findings.

First, the authors stratified patients according to baseline serum uric acid above the laboratory upper limit of normal—not according to a clinical diagnosis of hyperuricemia. This laboratory-based stratification is methodologically reasonable for risk enrichment, but it does not coincide with the thresholds that trigger urate-lowering therapy in routine practice. In the absence of gout, patients who start above the upper limit of normal are not, per se, candidates for pharmacologic urate lowering.^2^ However, because bempedoic acid predictably raises serum uric acid, a proportion of these individuals may subsequently cross concentrations associated with monosodium urate supersaturation and develop a new, treatment-worthy indication for xanthine oxidase inhibition once therapy is underway.

Moreover, 2 distinct pathophysiological dimensions of uric acid elevation should be recognized. Mild or moderate increases—below the saturation threshold—may still act as a chronic risk factor contributing to cardiovascular and renal disease progression, whereas higher concentrations predispose to urate crystal deposition and overt gout.^3^ The former condition may remain clinically silent yet metabolically significant; the latter represents a symptomatic state requiring therapeutic intervention. Distinguishing between these scenarios is crucial to properly interpreting the metabolic implications of bempedoic acid therapy.

Second, further clarification on the baseline and on-trial use of drugs with mild (eg, atorvastatin, fenofibrate, losartan) or moderate (eg, sodium-glucose cotransporter-2 inhibitors) uricosuric effects would be valuable, as they may have mitigated the apparent hyperuricemic effect of bempedoic acid.^4^

Third, it would be of interest to explore whether untreated uric acid elevations during bempedoic acid therapy were associated with any change in renal function. Renal complications linked to elevated uric acid often develop after prolonged exposure, particularly in individuals with preserved kidney function at baseline,^3^ and the duration of CLEAR Outcomes may not have been sufficient to detect such changes.^5^ Longer follow-up could therefore provide important information regarding the broader metabolic and renal safety profile of bempedoic acid.

Finally, because xanthine oxidase inhibition appears to mitigate gout risk in patients who develop uric acid elevations during therapy, future cost-effectiveness analyses might integrate the potential downstream costs of urate-lowering treatment, renal monitoring, and periodic uric acid testing. This would allow for a more comprehensive assessment of long-term management strategies accompanying bempedoic acid use.

We congratulate the authors on their thoughtful and clinically relevant contribution and hope that these reflections may further enrich the discussion on optimizing bempedoic acid therapy in clinical practice.
